# Epidemiology of Buruli Ulcer in Victoria, Australia, 2017–2022

**DOI:** 10.3201/eid3103.240938

**Published:** 2025-03

**Authors:** Bhavi Ravindran, Daneeta Hennessy, Miriam O’Hara, Ee Laine Tay, Rosalina Sa’aga Banuve, Jodie McVernon, Kylie Carville

**Affiliations:** Victorian Infectious Diseases Reference Laboratory, Royal Melbourne Hospital, at the Peter Doherty Institute for Infection and Immunity, Melbourne, Victoria, Australia (B. Ravindran, K. Carville); Australian National University, Canberra, Australian Capital Territory, Australia (B. Ravindran, R. Sa’aga Banuve); Department of Health, Melbourne (D. Hennessy, M. O’Hara, E.L. Tay); University of Melbourne Department of Microbiology and Immunology, at the Peter Doherty Institute for Infection and Immunity, Melbourne (J. McVernon, K. Carville)

**Keywords:** Buruli ulcer, Australia, Melbourne, Australia, Mycobacterium ulcerans, endemic, epidemiology, tuberculosis and other mycobacteria, bacteria

## Abstract

Buruli ulcer (BU) is a rare, neglected tropical disease caused by *Mycobacterium ulcerans* that can lead to severe skin ulcers. To determine the epidemiology of BU in Victoria, Australia, during 2017–2022 we analyzed surveillance data. A total of 1,751 cases of BU were notified; 968 (55%) patients were male and 781 (45%) female (2 were missing sex data), and 984 (56%) resided in established BU-endemic areas, although an increasing number were in new BU-endemic areas. Most cases (83%, 1,301) were classified as category I. Multivariate modeling demonstrated that factors for severe BU included being male, being older, and living in a new BU-endemic or non–BU-endemic area. A relatively shorter interval between first visit to a clinician and receipt of diagnosis was protective against severe disease. The expansion of BU-endemic areas throughout Victoria remains a public health concern and calls for targeted action, particularly for patients and clinicians in new BU-endemic areas.

Buruli ulcer (BU) is a devastating skin and tissue infection caused by *Mycobacterium ulcerans* (*1*). BU is prevalent mainly in tropical sub-Saharan Africa, although ≈30 countries have reported cases (*2*). Although the number of cases has decreased worldwide, local epidemics in Australia have countered that trend (*3*,*4*). BU-endemic regions in Australia include the Daintree Rainforest and the Capricorn region in tropical Queensland and the East Gippsland and metropolitan Greater Melbourne/Bellarine regions in the state of Victoria in southeastern Australia (*3*,*5*,*6*). The climate in the southeastern state of Victoria is temperate; temperature and weather vary substantially throughout the year (*7*).

BU is usually exhibited initially as a painless skin nodule that predominantly affects the distal limbs and, if left untreated, forms a characteristic ulcer with undermined edges (*8*). The average incubation period for BU is ≈4–5 months, and the average delay between symptom onset and diagnosis is 1–2 months (*9*). Although the BU mortality rate is low, the illness can result in substantial socioeconomic effects on individual persons and communities (*2*,*10*).

Residence in or visitation to a BU-endemic area remains a significant risk factor for *M. ulcerans* acquisition; previous BU outbreaks have occurred as geographically defined infections (*11*). In the temperate climates of Australia, transmission research has focused on mosquitoes as vectors and small Australia native marsupials (possums) as animal reservoirs (*12*,*13*). Mosquitoes are infected by biting possums that carry the bacteria, after which they directly inoculate humans, causing clinical disease (*13*,*14*). An environmental study has shown a correlation between rainfall and BU, as is seen for other vectorborne diseases in the region, including Barmah Forest and Ross River fevers, which further supports the role of mosquitoes (*15*). Definitive evidence was provided through an extensive field survey and genomic analysis that indicated that mosquitoes transmit *M. ulcerans* in southeastern Australia from a reservoir of possums (*16*).

BU was first identified in Victoria in 1948, and only 50 cases were recorded before 1990 (*17*). Since then, the pattern of disease has changed substantially, from low numbers in fixed geographic regions to more widespread transmission (*3*,*18*). New areas of endemicity have emerged, and cases have increased continually since 2011 (*11*,*19*). Within Melbourne, the emergence, continued propagation, and expansion of BU-endemic areas remains a public health concern (*10*).

Using routinely collected surveillance data, we analyzed the epidemiology of BU in Victoria during 2017–2022, identifying factors that influence disease severity and mapping the ongoing spread of the disease. Ethics approval was provided by the Australian National University Human Research Ethics Committee (protocol 2017/909).

## Methods

In Victoria, BU has been a notifiable condition since 2004; reporting has been required by laboratories and clinicians under the Public Health and Wellbeing Regulations of 2019 (*20*). The study population included all patients with confirmed cases notified to the Victoria Department of Health during 2017–2022.

### Data Sources

We obtained case data from the Public Health Event Surveillance System database of Victoria. Since January 1, 2011, the Victoria Department of Health has collected enhanced surveillance forms that are completed by notifying clinicians or by public health officers from case interviews. Information collected included patients’ date of birth, sex, residential address, history of travel to or residence in BU-endemic areas in the 12 months before symptom onset, date of symptom onset, date of first visit to a clinician, date when a clinician first suspected BU, form of the disease, size of the affected area (World Health Organization [WHO] categories I, II or III), lesion location, laboratory results (PCR or culture), and treatment details. To determine the rate of BU per 100,000 population, we obtained information about the population of Victoria from the Australian Bureau of Statistics (*21*).

### Definitions

Before July 2021, a confirmed case of BU was defined by definitive laboratory evidence of infection as either PCR detection of IS2404 insertion sequences or culture identification of *M. ulcerans* from a tissue specimen or lesion swab sample (*22*). After July 2021, a confirmed case was defined by definite laboratory evidence as above and by clinical evidence as a clinical diagnosis of BU made by a clinician experienced in the management of BU, including clinical follow-up to ensure a consistent clinical course (*22*).

**Figure 1:**
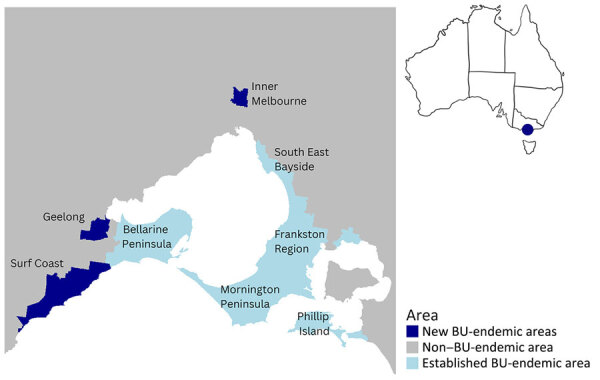
Geographic areas in Greater Melbourne and Bellarine region, Australia, highlighting new (Inner Melbourne, Geelong and Surf Coast), established (Mornington Peninsula, Bellarine Peninsula, South East Bayside, Frankston region, Philip Island), and non–BU-endemic areas, 2017–2022. Not shown: East Gippsland BU-endemic area, which is to the east of the state. BU, Buruli ulcer.

The Australia Department of Health has defined BU-endemic areas (*11*,*19*) as places where >2 residents had BU without recalled travel to another BU-endemic area in the previous year, places adjacent to an endemic area with >1 affected residents or visitors without recalled travel history, or places where *M. ulcerans* has been detected in the environment (*22*). We classified BU-endemic areas in Victoria into 3 categories: established BU-endemic, new BU-endemic, and non–BU-endemic areas. Established areas were Mornington Peninsula, Bellarine Peninsula, Phillip Island, East Gippsland, South Eastern Bayside suburbs, and Frankston region because they had been described in previous analyses (*11*,*19*). New BU-endemic areas included Surf Coast and Geelong (first identified in 2017) and Inner Melbourne (first identified in 2019) (Appendix). Non–BU-endemic areas were all other areas in Victoria not previously listed (Figure 1). The Department of Health recorded primary exposure as the most likely area of BU acquisition, considering the duration and frequency of exposure to known BU-endemic areas and exposure timing relative to symptom onset. If a case-patient resided in a BU-endemic area, the primary exposure was considered to be the patient’s home address, given the assumed duration and frequency of exposure. For case-patients who reported no history of residence in or travel to known BU-endemic areas, primary exposure was considered to be the home address at the time of diagnosis.

Lesion severity was classified according to WHO definitions (*23*). Category I comprises single, small lesions <5 cm in diameter; category II comprises single lesions of 5–15 cm in diameter; and category III comprises single extensive lesions >15 cm in diameter, multiple lesions, lesions at critical sites (e.g., eye, genitalia, joints), and osteomyelitis. Severe disease was classified as category II or category III lesions.

Similar to previous studies, delay to first visit was calculated as days from symptom onset to first visit to a healthcare practitioner (*19*). Diagnosis delay was days from first visit to a healthcare practitioner to diagnosis date, approximated by the date of notification to the Department of Health (*19*).

### Statistical Analyses

We imported de-identified data into R version 4.3.2 (The R Project for Statistical Computing, https://www.r-project.org) for analysis. To illustrate the study population, we descriptively analyzed data. We described first visit, diagnosis, and total delays by using the median and interquartile range. To explore differences between groups, we used χ^2^ or Fischer exacts tests for categorical variables and Kruskal-Wallis or Mann-Whitney U tests for continuous variables. We excluded cases from multivariate analysis if WHO lesion severity outcome, lesion location, or manifestation of BU was missing or if diagnosis or first visit delay could not be calculated because of missing information. We assessed risk factors of disease severity by using logistic regression between independent variables (patient sex, age, residential location at time of notification; first visit delay; and diagnosis delay) and the outcome variable of severe disease. We included area of residence, as opposed to primary exposure location, because that reflected where case-patients would access healthcare. We considered all independent variables for which univariate analysis indicated p<0.25 for inclusion in the multivariate model. To identify differences between included and excluded case-patients that were used in the final multivariate model, we conducted sensitivity analyses. 

## Results

During 2017–2022, a total of 1,751 confirmed cases of BU were notified to the Australia Department of Health (Table 1). More than half of the patients were male (968 [55%] male and 781 [45%] female; data on sex were missing for 2); most were 16–60 (883 [50%]) or >60 (721 [40%]) years of age. Approximately half of the patients lived in an established BU-endemic area (984 [56%]). The most common lesion location was the lower limbs (74%); a small number of patients had lesions at multiple sites (2%). Most lesions were category I (1,301 [83%]). After a drop in case numbers in 2020, case numbers in 2021 and 2022 were similar to those before the COVID-19 pandemic; case numbers for 2022 (334 [19% of notified cases in the study period]) were similar to the previous high number from 2018 (340 [19%]). Of the 1,604 patients for whom treatment was recorded, most patients received antimicrobial therapy alone as treatment (1,144 [71%]) followed by a combination of surgery and antimicrobial drug treatment (332 [21%]). The median time to seeking care was 28 days (95% CI 11–50 days), and the median time between seeking care and diagnosis was 19 days (95% CI 7–42 days). Of the 1,614 patients with a recorded manifestation, the most common manifestation was ulcers (1,227 [76%]) (Table 1). Of the 387 nonulcerous manifestations of BU, most common were cellulitis (126 [33%]), nodules (108 [28%]), and papules (89 [23%]).

**Table 1:** Characteristics of BU cases notified to the Victoria Department of Health, overall and by location of residence, Victoria, Australia, 2017–2022*

Variable	Overall, n = 1,751	BU area			p value
		New, n = 163	Non–BU-endemic, n = 604	Established, n = 984	
Sex					<0.05
F	781 (45)	78 (48)	244 (40)	459 (47)	
M	968 (55)	84 (52)	359 (60)	525 (53)	
Missing	2	1	1	0	
Age group, y					<0.001
0-15	148 (8.5)	16 (9.8)	58 (9.6)	74 (7.5)	
16-60	883 (50)	90 (55)	352 (58)	441 (45)	
>60	720 (41)	57 (35)	194 (32)	469 (48)	
Lesion location					<0.05
Arm	350 (21)	41 (27)	101 (17)	208 (23)	
Leg	1,206 (74)	103 (68)	460 (78)	643 (71)	
Multiple	37 (2.3)	4 (2.6)	15 (2.6)	18 (2.0)	
Other	47 (2.9)	4 (2.6)	12 (2.0)	31 (3.4)	
Missing	111	11	16	84	
WHO lesion category					<0.05
I	1,301 (83)	114 (77)	462 (81)	725 (85)	
II	176 (11)	21 (14)	77 (13)	78 (9.2)	
III	95 (6.0)	14 (9.4)	32 (5.6)	49 (5.8)	
Missing	179	14	33	132	
Year of notification					0.001
2017	277 (16)	12 (7.4)	110 (18)	155 (16)	
2018	340 (19)	10 (6.1)	125 (21)	205 (21)	
2019	299 (17)	19 (12)	115 (19)	165 (17)	
2020	217 (12)	16 (9.8)	69 (11)	132 (13)	
2021	284 (16)	41 (25)	78 (13)	165 (17)	
2022	334 (19)	65 (40)	107 (18)	162 (16)	
Treatment					0.001
Antibiotics	1,144 (71)	111 (73)	386 (66)	647 (75)	
Antibiotics and surgery	332 (21)	34 (22)	159 (27)	139 (15)	
Other	90 (5.6)	6 (3.9)	21 (3.6)	63 (7.3)	
Surgical	38 (2.4)	1 (0.7)	16 (2.7)	21 (2.4)	
Missing	147	11	22	114	
Diagnosis delay, d, median (IQR)	19 (7–42)	22 (9–44)	31 (13–58)	13 (6–31)	<0.001
Missing	209	20	48	141	
Presentation d, median, (IQR)	28 (11–50)	27 (9–45)	30 (14–61)	24 (10–47)	0.001
Missing	251	21	60	170	
Manifestation					<0.05
Nonulcer	387 (24)	50 (33)	129 (22)	208 (24)	
Ulcer	1,227 (76)	102 (67)	454 (78)	671 (76)	
Missing	137	11	21	105	

*Values are no. (%) except as indicated. Percentages exclude missing data. BU, Buruli ulcer; IQR, interquartile range; WHO, World Health Organization.

The overall rate of BU diagnosis in Victoria was 4.48 cases/100,000 population during the study period. The lowest annual rate was 3.28 cases/100,000 population in 2020, and the highest rate was 5.29 cases/100,000 population in 2018.

### Demographic Differences by Area of Residence

We found significant differences in sex, age grouping, WHO severity score, diagnosis delay, manifestation delay, and manifestation between residents in new, established, and non–BU-endemic areas. Compared with new and non–BU-endemic areas, case-patients residing in established areas were more likely to be older (48% >60 years of age in established areas, 32% in non–BU-endemic areas, 35% in new areas; p<0.001); to have category I disease (85% in established areas, 81% in non–BU-endemic areas, 77% in new areas; p = 0.006); to have a shorter diagnosis delay (p<0.001) and shorter delay before first visit (p<0.001) (Table 1). The location of lesions also differed; case-patients in non–BU-endemic areas were more likely than those in other areas to have a lesion on their lower limbs (78% in non–BU-endemic areas, 71% in established areas, and 68% in new areas; p = 0.027) and more likely to have different treatments recorded at the time of public health follow-up visits (66% received antimicrobial drugs in non–BU-endemic areas, 75% in established areas, and 73% in new areas; p = 0.027) (Table 1).

When compared with non–BU-endemic and established areas, case-patients in new BU-endemic areas were more likely to be notified in 2021 and 2022 (p<0.001) and to not have an ulcer (33% in new, 24% in established, and 22% in non–BU-endemic areas; p = 0.022) The proportions of male and female case-patients differed by area of residence (p = 0.036) (Table 1).

### Demographic Differences by Age and Sex

With respect to age, we noted significant differences in lesion severity, area of residence, and first visit delay. Patients >60 years of age were more likely to have category II or category III ulcers (19% of patients >60 years of age, 16% of patients 16–60 years of age, 17% of patients 0–15 years of age; p = 0.002) and to live in an established BU-endemic area (65% of patients >60 years of age, 50% of patients 16–60 years of age, 50% of patients 0–15 years of age; p<0.001) (Table 2). Patients who were 16–60 years of age were more likely to have a longer delay to first visit (p<0.001) and to have received more antimicrobial drugs without surgery (p = 0.01) than were patients who were older and younger. We found no significant differences by age group in terms of sex, year of diagnosis, diagnosis delay, or manifestation type (Table 2).

**Table 2:** Characteristics of BU cases notified to the Victoria Department of Health, by age group and sex, Victoria, Australia, 2017–2022*

Variable	Age group, y					Sex		
	No.	0–15, n = 148	16–60, n = 883	>60, n = 720		No.	F, n = 781	M, n = 968
Sex	1,749							
F		69 (47)	378 (43)	334 (46)			NA	NA
M		78 (53)	504 (57)	386 (54)			NA	NA
Missing		1	1	0				
Lesion location	1,640					1,638		
Upper limb		19 (13)	147 (18)	184 (27)			169 (23)	181 (20)
Lower limb		120 (84)	628 (76)	458 (68)			524 (72)	680 (75)
Multiple		2 (1.4)	21 (2.5)	14 (2.1)			12 (1.6)	25 (2.8)
Other		2 (1.4)	28 (3.4)	17 (2.5)			24 (3.3)	23 (2.5)
Missing		5	59	47			52	59
WHO lesion category	1,572 (p<0.01)					1,570 (p<0.05)		
I		116 (83)	671 (84)	514 (81)			599 (85)	701 (81)
II		20 (14)	88 (11)	68 (11)			71 (10)	105 (12)
III		3 (2.2)	36 (4.5)	56 (8.8)			32 (4.6)	62 (7.1)
Missing		9	88	82			79	100
Area of residence	1,751 (p<0.001)					1,749 (p<0.05)		
New		16 (11)	90 (10)	57 (7.9)			78 (10.0)	84 (8.7)
Non–BU-endemic		58 (39)	352 (40)	194 (27)			244 (31)	359 (37)
Established		74 (50)	441 (50)	469 (65)			459 (59)	525 (54)
Year	1,751					1,749		
2017		27 (18)	131 (15)	119 (17)			132 (17)	143 (15)
2018		31 (21)	173 (20)	136 (19)			142 (18)	198 (20)
2019		32 (22)	149 (17)	118 (16)			122 (16)	177 (18)
2020		11 (7.4)	102 (12)	104 (14)			94 (12)	123 (13)
2021		24 (16)	159 (18)	101 (14)			130 (17)	154 (16)
2022		23 (16)	169 (19)	142 (20)			161 (21)	173 (18)
Treatment	1,604 (p<0.05)					1,602		
Antibiotics		99 (70)	600 (75)	445 (67)			494 (70)	650 (73)
Both		37 (26)	141 (18)	154 (23)			146 (21)	184 (21)
Dressings/other		5 (3.5)	43 (5.4)	42 (6.4)			43 (6.1)	47 (5.3)
Surgical		0 (0)	19 (2.4)	19 (2.9)			24 (3.4)	14 (1.6)
Missing		7	80	60			74	73
Diagnosis delay, d, median (IQR)	1,542	17 (10–36)	21 (8–46)	16 (7–38)		1,540 (p<0.01)	20 (8–44)	17 (7–42)
Missing		17	115	77			92	117
First visit delay, d, median (IQR)	1,500 (p<0.001)	22 (12–35)	30 (14–60)	21 (7–45)		1,498	28 (9–54)	28 (13–48)
Missing		18	134	99			109	142
Manifestation	1,614					1,612 (p<0.001)		
Nonulcer		40 (28)	177 (22)	170 (26)			203 (28)	184 (21)
Ulcer		101 (72)	633 (78)	493 (74)			514 (72)	711 (79)
Missing		7	73	57			64	73

*Values are no. (%) except as indicated. Percentages exclude missing data. BU, Buruli ulcer; IQR, interquartile range; NA, not applicable; WHO, World Health Organization.

With respect to patient sex, we found significant differences in lesion category, area of residence, diagnosis delay, and having an ulcer compared with other manifestations. Male patients were more likely than female patients to have category II or category III ulcers (7.1% of lesions in male vs. 4.6% of lesions in female patients were category III; p = 0.036); reside in different areas (p = 0.036); have a shorter delay to diagnosis (p = 0.06); or have an ulcer (79% male vs. 72% female; p<0.001) (Table 2). We found no significant differences by sex between age group, lesion location, area of residence, treatment, or delay to first visit (Table 2).

### Notifications by Residence

**Figure 2:**
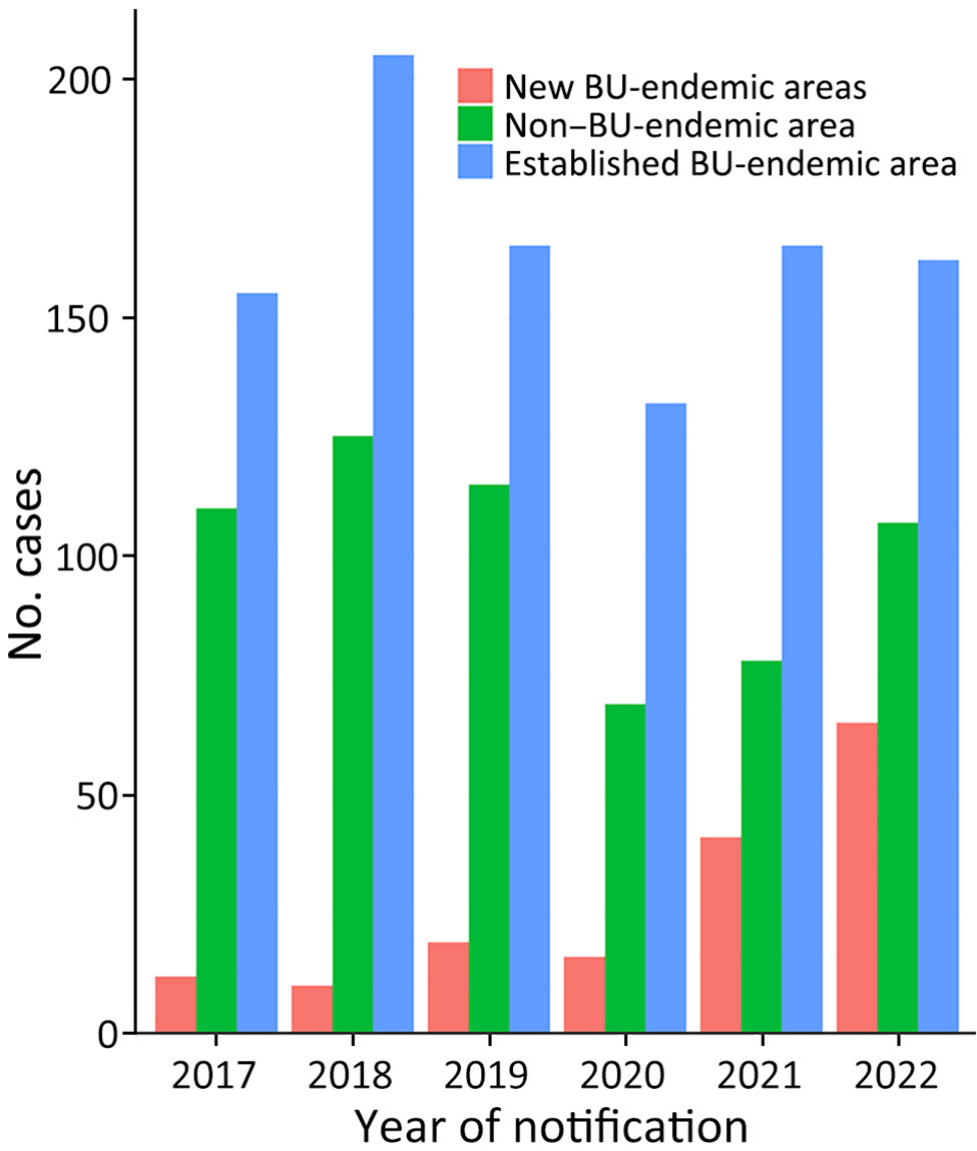
Cases of BU notified to the Victoria Department of Health, by area of residence and year, Victoria, Australia, 2017–2022. BU, Buruli ulcer.

Patient places of residence, by endemicity classification, were similar during 2017–2019. In 2020, the proportion of cases from non–BU-endemic areas dropped substantially. Patients residing in new BU-endemic areas increased relative to non–BU-endemic and established areas from 2020 (7% [16/217] of patients to 19% [65/334] of patients in 2022) (Figure 2). The increased cases in the new BU-endemic areas primarily resulted from patients residing in the inner Melbourne BU-endemic area.

### Notifications by Primary Exposure Location

**Figure 3:**
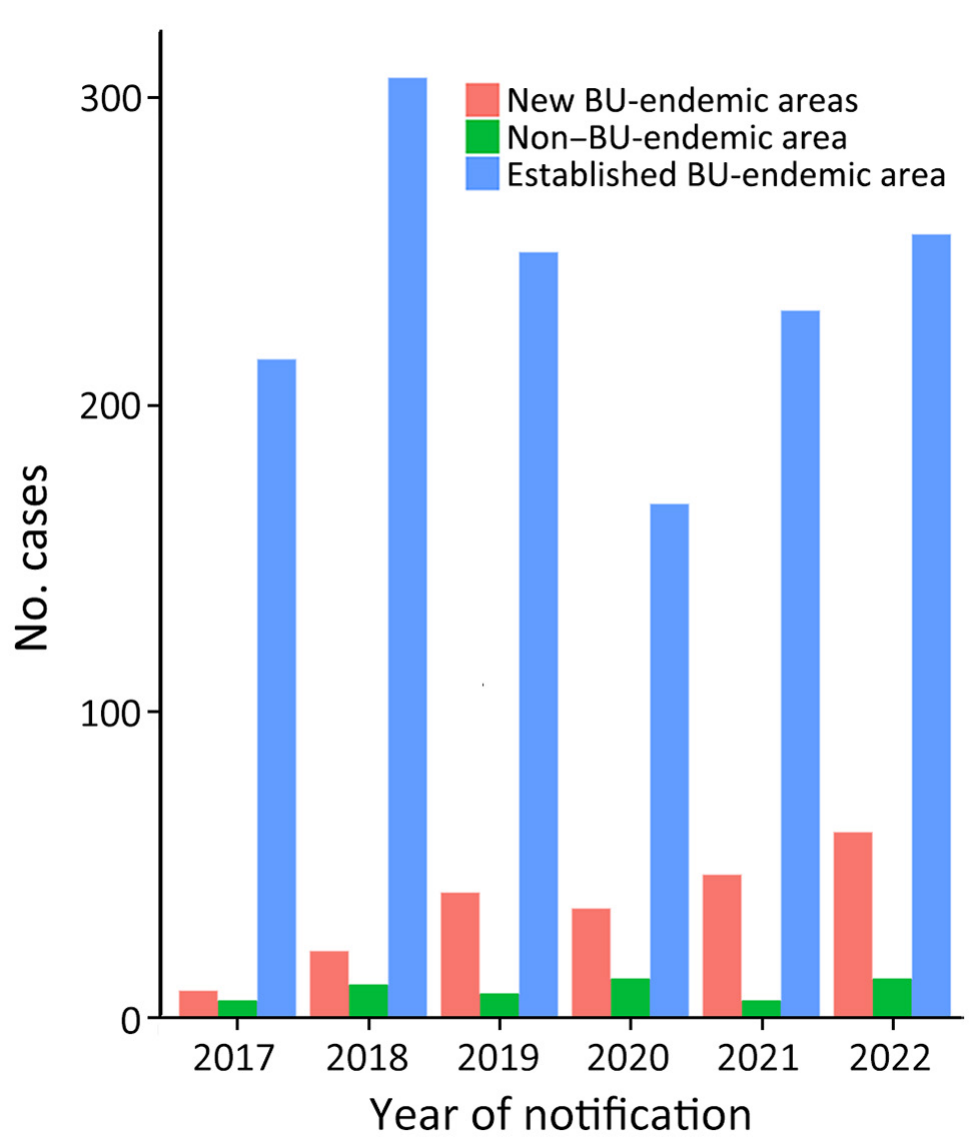
BU primary exposure locations, by region and year, Victoria, Australia, 2017–2022. BU, Buruli ulcer.

Primary exposure location was available for 1,700 (97.1%) case-patients. Over the study period, the most common primary exposure area continued to be established areas (1,427 [84%]), followed by new BU-endemic areas (216 [13%]) and then non–BU-endemic areas (57 [3%]). The proportion of patients whose primary exposure location was a new BU-endemic area increased substantially from 2017 to 2022 (4% [9/230] of exposures to 18% [61/330] of exposures), mirrored by a decrease in a primary exposure location in established endemic areas (93% [215/230] of exposures to 78% [256/330] of exposures (Figure 3).

**Figure 4:**
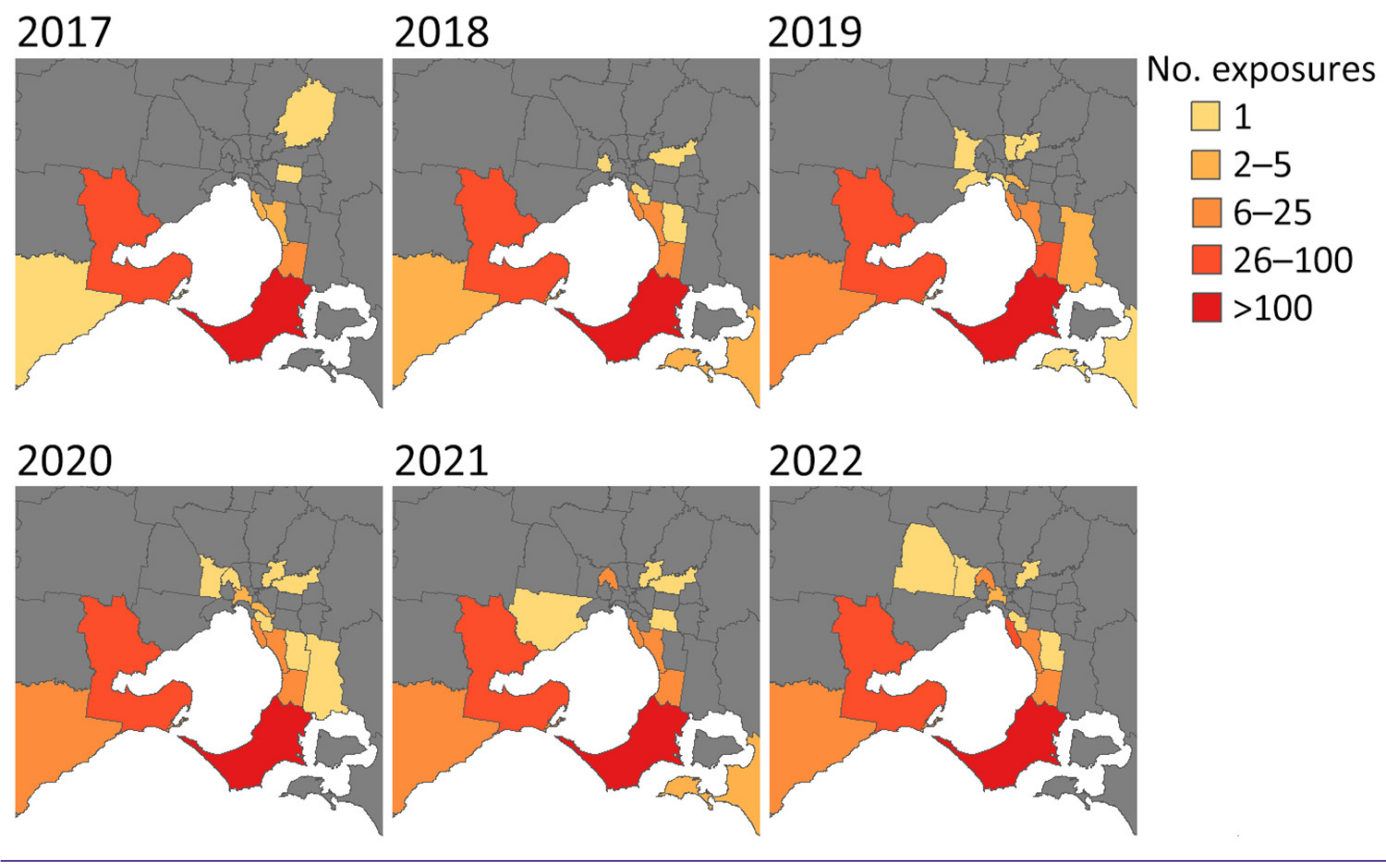
Change in Buruli ulcer primary exposures areas in Greater Melbourne and Bellarine region over time by local government area, Victoria, Australia, 2017–2022.

During the study period, the higher number of primary exposures were in the Mornington Peninsula (1,028 [60%]), followed by the Bellarine Peninsula (223 [13%]) and the Frankston Area (116 [7%]). For 57 (3%) patients, no travel to BU-endemic areas was reported (Figure 4). Although still relatively low compared with the Mornington Peninsula, of note is the emergence of the inner-city Melbourne area, in which primary exposure locations substantially increased in from 2019 (0 exposures) to 2022 (37 exposures, 11%) (Figure 4).

### Seasonality

**Figure 5:**
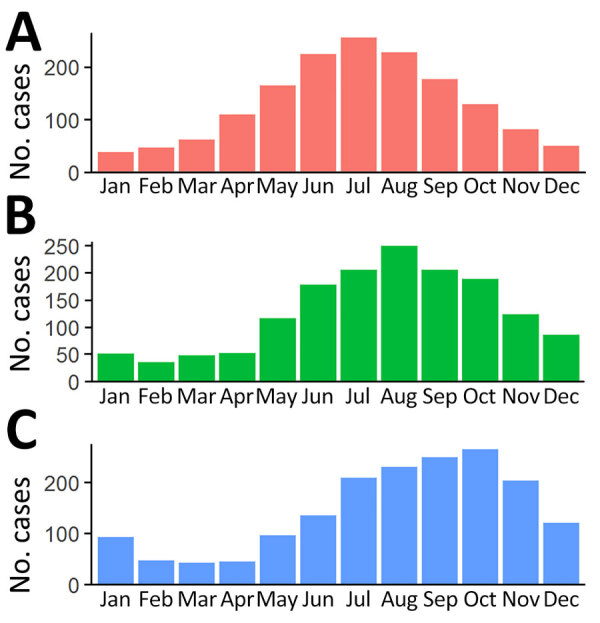
Timing of symptom onset (A), first visit to a clinician (B), and notification of Buruli ulcer (C) among cases notified to Victoria Department of Health, Victoria, Australia, 2017–2022.

The date of symptom onset was available for 1,573 (89.8%) patients, and the date of first visit was available for 1,541 (88.1%) of patients. Symptom onset was most frequent in July (winter in Victoria) and least often in January (summer in Victoria). The peak for healthcare visitation was August, and the peak for BU diagnosis was October (Figure 5).

### Risk Factors for Severe Disease

Of the 1,751 cases, we excluded 357 (20%) from the regression model because information was missing for either the dependent variable; WHO lesion category; or the independent variables sex, delay to first visit, delay to diagnosis, or manifestation. Included patients were less likely to be from an established BU-endemic area (p<0.001), have a longer delay to diagnosis where recorded (p<0.001), and to have been notified in 2021 (p<0.001). We found no differences in sex, age grouping, delay to first visit, manifestation with an ulcer, or lesion category (Table 3).

**Table 3:** Characteristics of BU cases notified to the Victoria Department of Health, by exclusion or inclusion from logistic regression, Victoria, Australia, 2017–2022*

Variable	Excluded, n = 357	Included, n = 1,394	p value
Sex			0.3
F	149 (42)	632 (45)	
M	206 (58)	762 (55)	
Unknown	2	0	
Age, y, continuous	54 (35,70)	53 (36,69)	0.5
Area of residence			<0.001
New BU-endemic	30 (8.4)	133 (9.5)	
Non–BU-endemic	91 (25)	513 (37)	
Established BU-endemic	236 (66)	748 (54)	
Year			<0.001
2017	56 (16)	221 (16)	
2018	56 (16)	284 (20)	
2019	53 (15)	246 (18)	
2020	55 (15)	162 (12)	
2021	83 (23)	201 (14)	
2022	54 (15)	280 (20)	
Diagnosis delay, d, median (IQR)	13 (5, 33)	20 (8, 43)	<0.001
Unknown	209	0	
Presentation delay, d, median (IQR)	21 (2, 60)	28 (12, 50)	0.2
Unknown	251	0	
WHO lesion category			
I	145 (83)	1156 (81)	0.2
II/III	33 (17)	238 (19)	
Unknown	179	0	
*Values are no. (%) except as indicated. Percentages exclude missing data. BU, Buruli ulcer; WHO, World Health Organization.			

Multivariate regression revealed increased odds of severe BU disease among male patients (odds ratio [OR] 1.44 [95% CI 1.08–1.94]; p = 0.014) with increasing age per year (OR 1.01 [95% CI 1.00–1.01]; p = 0.015), residence in a new area (OR 1.69 [95% CI 1.02–2.73]; p = 0.035) or non–BU-endemic area (OR 1.38 [95% CI 1.01–1.88]; p = 0.042), or a longer delay to diagnosis per day (OR 1.00 [95% CI 1.00–1.01]; p<0.001) (Table 4). Notifications received in 2018 (OR 0.61 [95% CI 0.39–0.97]; p = 0.036) and 2021 (OR 0.41 [95% CI 0.23–0.71]; p = 0.002) were associated with significantly less severe disease than were cases notified in 2017.

**Table 4:** Univariate and multivariate associations between risk factors and severity of BU, Victoria, Australia, 2017–2022*

Variable	WHO severity			Univariate analysis					Multivariate analysis	
	I, n = 1,156	II/III, n = 238		OR	95% CI	p value	aOR		95% CI	p value
Sex										
F	541 (47)	91 (38)		1.00	NA	NA	1.00		NA	NA
M	615 (53)	147 (62)		1.42	1.07–1.90	0.016	1.44		1.08–1.94	0.014
Age, y, continuous	53 (35–69)	56 (41–74)		1.01	1.00–1.01	0.021	1.01		1.00–1.01	0.015
BU-endemic area										
New BU-endemic	105 (9.1)	28 (12)		1.56	0.97–2.46	0.059	1.69		1.02–2.73	0.035
Non–BU-endemic	416 (36)	105 (44)		1.44	1.07–1.94	0.017	1.38		1.01–1.88	0.042
Established BU-endemic	635 (55)	105 (44)		1.00	NA	NA	1.00		NA	NA
Year										
2017	171 (15)	50 (21)		1.00	NA	NA	1.00		NA	NA
2018	241 (21)	43 (18)		0.61	0.39–0.96	0.032	0.61		0.39–0.97	0.036
2019	203 (18)	43 (18)		0.72	0.46–1.14	0.2	0.68		0.43–1.09	0.11
2020	132 (11)	30 (13)		0.78	0.46–1.28	0.3	0.71		0.42–1.18	0.2
2021	179 (15)	22 (9.2)		0.42	0.24–0.72	0.002	0.41		0.23–0.71	0.002
2022	230 (20)	50 (21)		0.74	0.48–1.15	0.2	0.69		0.44–1.09	0.11
Diagnosis delay, d	18 (7–41)	28 (12–57)		1.03	1.01–1.04	<0.001	1.00		1.00–1.01	<0.001
First visit delay, d	28 (12–51)	26 (10–40)		1.00	1.00–1.00	0.7	NA		NA	NA

*Values are no. (%) except as indicated. aOR, adjusted odds ratio; BU, Buruli ulcer; NA, not applicable; OR, odds ratio; WHO, World Health Organization.

## Discussion

The continued increase of BU cases in Victoria demonstrates BU progression from a localized disease in small geographic clusters to further expansion and emergence of endemic areas (*24*). Although the Geelong and Surf Coast regions are contiguous to the well-established BU-endemic area of the Bellarine Peninsula, the inner Melbourne area is not coastal and shares no boundaries with known BU-endemic areas.

The expansion of BU-endemic areas is a public health concern, and monitoring the emergence of new areas is still needed. Current research provides evidence for possums as a reservoir and mosquitoes as vectors; thus, environmental surveillance through possum fecal excreta and mosquito surveys with screening for *M. ulcerans* may help supplement current activities for monitoring spread (*16*,*25*). *M. ulcerans* is probably introduced into new environments and then expands rather than emerging from a dormant pathogen reservoir; however, initial *M. ulcerans* introduction into new BU-endemic areas is unclear (*26*).

Demographics, clinical signs, and diagnosis delays differ by area of residence. Patient and clinician understanding of BU disease in established areas may be greater than that in new or non–BU-endemic areas, particularly with respect to care seeking and consideration of treatment options.

Similar to previous work, our study demonstrates that older age (*11*,*18*) and living in a new or non–BU-endemic area are associated with severe BU lesions (*11*). Of note, the multivariate model demonstrated that diagnosis delay, and not first visit delay, was associated with severe disease. Median delays between first visit (3 weeks) and diagnosis (4 weeks) in Victoria are considerably shorter than in other settings such as Nigeria (median delay of 29 weeks) and Cameroon (median delay of 12 weeks) (*27*,*28*). Factors that contributed to a longer delays in first visit and diagnosis in those countries include geography and inaccessibility to healthcare, which are unlikely to be factors in Victoria (*27*).

The temporal relationship between symptom onset, first visit, and notification of BU followed the previously described seasonal patterns: symptom onset peaking mid-winter and dipping mid-summer (*29*). The mosquito season in Victoria is November–April, which, given the median incubation period of 4–5 months, supports acquisition during the summer in Victoria (*9*). Therefore, targeted messages to the public in the warmer months with regard to prevention and patients and clinicians in the autumn/winter months with regard to early disease recognition and diagnosis should be strengthened (*30*).

Our study period encompasses the COVID-19 pandemic, and the effect of nonpharmaceutical control measures on transmission and public health follow-up was apparent. The state of Victoria experienced prolonged lockdowns and movement restrictions (*31*), which resulted in a low number of BU cases in 2020 and exclusion of several cases in 2021 from the regression model because of missing data. Competing public health priorities meant that BU patient follow-up could not always be consistently performed during that period.

Before the COVID-19 pandemic, we observed a lower proportion of patients with severe disease in 2018, possibly associated with increased public messaging within established areas (*32*). Another effect of the COVID-19 pandemic was the increased proportion of patients with severe disease in 2020. However, further public health messaging may have improved awareness among the public and clinicians, particularly in newly identified areas, and resulted in reduction of severe disease in 2021 (*33*).

Among the strengths of our study is inclusion of the extensive public health surveillance database. Limitations included exclusion of cases because of missing information. Because excluded case-patients were more likely to live in a BU-endemic area, have a shorter delay to diagnosis, and be notified in 2020 and 2021, disease might have been less severe for those case-patients, which might have biased the multivariate model to show a stronger association between independent variables and severe BU lesions. Furthermore, because the data were from a notifiable public health disease database, data on other factors that could have influenced the severity of disease were not available, including medical comorbidities, socioeconomic status, or access to healthcare facilities. Last, primary exposure information was not collected consistently across established, new, or non–BU-endemic areas over the study period, potentially resulting in misclassification.

Our study findings contribute to the substantial body of work on BU in Victoria. However, several findings are concerning, including the near tripling of cases during 2017–2022 compared with 2011–2016 and the emergence of multiple new BU-endemic areas (*11*). The continued propagation and increased case numbers call for clear, targeted, and effective public health action, which may include continued surveillance of human cases, enhanced surveillance of mosquito and possum excreta, mosquito control activities, public health messaging, and clinician education.

Appendix Additional information for epidemiology of Buruli ulcer in Victoria, Australia, 2017–2022.
