# Supplementary material for: Epidemiology of Buruli Ulcer in Victoria, Australia, 2017–2022
Source: Emerg Infect Dis. 2025 Mar;31(3):448–57. doi: 10.3201/eid3103.240938 (PMC11878321; doi:10.3201/eid3103.240938)
Supplement: Appendix — Additional information for epidemiology of Buruli ulcer in Victoria, Australia, 2017–2022. [file 24-0938-Techapp-s1.pdf]

Article DOI: https://doi.org/10.3201/eid3103.240938

EID cannot ensure accessibility for supplementary materials supplied by authors. Readers who have difficulty accessing supplementary content should contact the authors for assistance.

Epidemiology of Buruli Ulcer in Victoria, Australia, 2017–2022

Appendix

Appendix Table. Current List of Affected Buruli ulcer Endemic Area in Victoria

Local Government Area (LGA)	Suburbs or towns in LGA	Postcodes	Endemic Area
Mornington Peninsula (S)	Arthurs Seat, Balnarring, Balnarring Beach, Bittern, Blairgowrie, Boneo, Cape Schanck, Capel Sound, Crib Point, Dromana, Fingal, Flinders, Hastings, HMAS Cerberus, Main Ridge, McCrae, Merricks, Merricks Beach, Merricks North, Moorooduc, Mornington, Mount Eliza, Mount Martha, Pearcedale (part), Point Leo, Portsea, Red Hill, Red Hill South, Rosebud, Rye, Safety Beach, Shoreham, Somers, Somerville, Sorrento, St Andrews Beach, Tootgarook, Tuerong, Tyabb	3912, 3913, 3915, 3916, 3918, 3919, 3920, 3926, 3927, 3928, 3929, 3930, 3931, 3933, 3934, 3936, 3937, 3938, 3939, 3940, 3941, 3942, 3943, 3944	Mornington peninsula
Westernport region	Tooradin, Warneet	3980	Mornington peninsula
Frankston area	Carrum Downs, Frankston, Frankston North, Frankston South, Langwarrin, Langwarrin South, Seaford, Sandhurst, Skye	3198, 3199, 3200, 3201, 3910, 3911	Frankston area
Bayside (C)	Beaumaris, Black Rock, Brighton, Brighton East, Cheltenham (part), Hampton, Hampton East, Highett (part), Sandringham	3186, 3187, 3188, 3190, 3191, 3192, 3193,	Melbourne South East suburbs
Kingston (C)	Aspendale, Aspendale Gardens, Bonbeach, Braeside, Carrum, Chelsea, Chelsea Heights, Cheltenham (part), Edithvale, Highett (part), Mentone, Mordialloc, Parkdale, Patterson Lakes, Waterway	3194, 3195, 3196, 3197	Melbourne South East suburbs
Greater Geelong (C)	Includes Barwon Heads, Connewarre, Clifton Springs, Curlewis, Drysdale, Indented Heads, Leopold, Ocean Grove, Portarlington, St Leonards	3222, 3223, 3224, 3226, 3227	Bellarine Peninsula
Greater Geelong (C)	Belmont, Highton, Newtown, Wandana Heights, Grovedale, Marshall	3216, 3220	Geelong
Queenscliff (C)	Point Lonsdale, Queenscliff	3225	Bellarine Peninsula
Surf Coast (C)	Coastal towns from Breamlea and Aireys Inlet. Includes Aireys Inlet, Anglesea, Breamlea, Jan Juc, Moggs Creek, Torquay	3227, 3228, 3230, 3231	Surf Coast
Moreland (C)	Brunswick West, Pascoe Vale South	3055, 3044	Inner Melbourne
Moonee Valley (C)	Essendon, Moonee Ponds, Strathmore	3039, 3040, 3041	Inner Melbourne
Philip Island	Includes Cowes, Ventnoor, Silverleaves	3922	Philip Island
East Gippsland (S)	Coastal towns/areas from East of Sale to border of NSW. Includes Bairnsdale, Cape Conrarn, Lakes Entrance, Mallacoota, Metung, Nicholson, Swan Reach	3851, 3862, 3875, 3878, 3880, 3882, 3885, 3886, 3887, 3888, 3889, 3890, 3891, 3892, 3895, 3902, 3903, 3904, 3909	East Gippsland
